# Perceptions of Psychological Intimate Partner Violence: The Influence of Sexual Minority Stigma and Childhood Exposure to Domestic Violence among Bisexual and Lesbian Women

**DOI:** 10.3390/ijerph18105356

**Published:** 2021-05-18

**Authors:** Sabrina Islam

**Affiliations:** 1 School of Public Health, University of California, 2121 Berkeley Way, Berkeley, CA 94720, USA; sislam@berkeley.edu; 2 Prevention Research Center, Pacific Institute for Research and Evaluation, 2150 Shattuck Avenue, Suite 601, Berkeley, CA 94704, USA

**Keywords:** intimate partner violence, stigma, sexual minority women, bisexual, lesbian, domestic violence, psychological

## Abstract

Sexual minority women (SMW; bisexual, lesbian) experience psychological intimate partner violence (IPV) disproportionately more than physical forms and have higher lifetime victimization rates than heterosexual women. This study presents an examination of perceptions of psychological IPV, sexual minority stigma, and childhood exposure to domestic violence among a sample of 183 SMW residing within the U.S. With an emphasis on group differences between bisexual and lesbian women, findings indicate that bisexual women evaluated vignettes depicting psychological IPV occurring among women in same-gender relationships with more negative sentiment than lesbian women. Significant associations between enacted and internalized forms of stigma and perceptions of psychological IPV also varied among bisexual and lesbian women. No significant relationships were found between perceived stigma and perceptions of IPV in either group. Furthermore, no moderation effects were detected for childhood exposure to domestic violence or sexual orientation in the relationship between sexual minority stigma and perceptions of IPV. Implications and suggestions are discussed with particular attention to the heterogeneity of experiences among SMW as a necessary area of further study.

## 1. Introduction

Intimate partner violence (IPV) is a preventable, widespread public health crisis that disproportionately harms women [1]. In the United States, over one in three women will experience IPV in their lifetime [2]. When grouping women by sexual orientation, prevalence rates reveal that the burden of IPV is even higher in sexual minority women (SMW; bisexual, lesbian) compared with heterosexual women. Over 60% of bisexual women and nearly 40% of lesbian women will be harmed by an intimate partner [3]. These elevated risks cannot be fully understood without acknowledging the under-representation of same-gender relationships in society. Dominant sociocultural norms simultaneously accept heterosexual relationships and reject same-gender relationships; such values may play an important role in how people perceive aspects of intimate relationships, including IPV [4,5]. The stigma experienced by bisexual and lesbian women may have a damaging influence on healthy partnerships [4]. The exposure to harm that can occur in same-gender relationships from IPV may be compounded by the harm of sexual minority stigma occurring outside of intimate relationships [6]; a cumulative effect may further isolate bisexual and lesbian women and compel them to tolerate IPV especially when it takes a nonphysical form. The cost of tolerance poses a major risk to safety as it produces confusion about defining violence [7]; the risk to safety may also be a product of the existing inequities and other related forms of exclusion experienced by SMW outside of the intimate relationship; namely, bisexual and lesbian women encounter the same IPV barriers as heterosexual women, but they are further disadvantaged by the stigma attached to their sexual identities, attractions, and relationships [8].

The lack of consensus in psychological IPV definitions [9] may reflect the ambiguity attached to this type of abuse, and therefore, positions of ambivalence may be strengthened and complicate collective agreement that psychological IPV be treated as harmful. Despite this, studies on psychological IPV have been instrumental in determining prevalence rates that are comparable to and higher than physical and sexual forms of IPV [1,9,10]. Research has also highlighted that, among women experiencing IPV, those who indicated the presence of psychological IPV were more likely to report adverse mental and physical health outcomes compared with women who reported only physical forms of IPV victimizations [11]. In disentangling the health implications of psychological IPV, other works have underscored victim-centered approaches to illuminate that psychological IPV incurred more suffering than physical IPV, and among these study samples, such women also showed higher levels of shame, fear, and mental distress [12]. The vast majority of such scholarships have been limited to a heteronormative framing of IPV, necessitating a centering of bisexual and lesbian women and their IPV experiences.

A comprehensive understanding of IPV attests to all forms of power and control. Through verbal and nonverbal tactics, psychological IPV relies on manipulation, deception, shame, intimidation, isolation, and coercion [13,14,15], which may also extend to the harassment, discrimination, and prejudice enacted against bisexual and lesbian women outside of a relationship. Hence, the scope of psychological IPV is complex for SMW. These overlapping forms of victimization call for greater attention to the multiple forms of sexual minority stigma within IPV studies of bisexual and lesbian women.

Existing research on psychological IPV has largely focused on health consequences and behavioral correlates [3,4,16]. In comparison, there have been fewer studies on how psychological IPV is viewed and even less examination of how those perceptions may be related to sexual minority stigma. Given a public sentiment of uncertainty surrounding psychological IPV, there may be a tendency for oppressed individuals and groups to minimize the harm they may experience because of their diminished status [17]. Germane works from social exchange theorists illuminate the negotiations and valuations that individuals attribute to their perceptions and decision-making [18,19]. As a result of their socially ascribed stigmatized statuses, bisexual and lesbian women may have fewer alternatives for help-seeking and support. Thus, it is imperative to explore how sexual minority women may specifically be driven to tolerate psychological IPV when it may be congruent with the hostility that they face in their neighborhoods, workplaces, communities, and greater society.

There has been limited research into the factors that influence permissive perceptions of IPV [20], especially among bisexual and lesbian women. Social-ecological and minority stress models offer a theoretical framework that accounts for factors operating at and above the individual levels as well as group-specific stressors and general life stressors occurring within a heterosexist context [5,21,22]. Sexual minority stress has been modelled through distal (e.g., perceived and enacted forms from others) and proximal stressors (e.g., directing external forms at the self through internalization) that create a climate of perpetual stigmatization [5]. Deteriorating effects of sexual minority stigma on both physical and mental health have been consistently found across varied sexual minority samples [5,23,24,25]. While the underlying processes are not yet clear, evaluations of internalized sexual minority stigma among bisexual and lesbian women have shown its significant effects on IPV victimization [4,26,27]. Parallel examinations of external sexual minority stigma, while limited, have yielded mixed findings with discrimination as the leading measure in IPV studies [4,28]. It remains possible that perceived and enacted forms of external sexual minority stigma are potent subtypes to consider.

To extend the body of knowledge that has demonstrated linkages of sexual minority stress to behavioral correlates of IPV [4,27,29,30], this study examines the relationships of well-documented risk factors and, for SMW and women in general, sexual minority stigma and childhood exposure to domestic violence. Within the trauma literature, specific attention to adverse experiences occurring in childhood has shown that an accumulation of abuse, neglect, and dysfunction within the household poses profound risks to health across the lifespan [31,32,33]. Witnessing and direct victimization of domestic violence is not uncommon and may heighten risks for future IPV, including perpetration and victimization [33,34,35,36]. The negative impacts of such experiences may be intensified in bisexual women, whose lives are disrupted by the dual sources of stigma from heterosexism and monosexism [37,38,39]. Given monolithic assumptions of stigma that eclipse the existence of variations among SMW, an exploration of group differences may be central to addressing the pervasive nature of violence within the lives of bisexual and lesbian women.Overall, the IPV literature has importantly focused on behavior; however, this may have the unintended effect of suggesting that IPV is not a problem until a person perpetrates or is victimized. Therefore, the current investigation of perceptions had the following goals:

1. To assess the association between sexual minority stigma and permissive perceptions of psychological IPV and how they differ by sexual orientation.
2. To examine the extent to which childhood exposure to domestic violence moderates the relationship between sexual minority stigma and permissive perceptions of psychological IPV.
3. To examine the extent to which sexual orientation moderates the relationship between sexual minority stigma and permissive perceptions of psychological IPV.

## 2. Materials and Methods

### 2.1. Procedures

A cross-sectional online questionnaire was accessed by a sample drawn from Qualtrics Panels, a crowdsourcing tool for obtaining survey responses [40]. Eligibility to participate was limited to individuals who self-identified as a bisexual or lesbian woman, were 18 years of age or older, and lived in the United States. These criteria were used to target potential participants with matching sociodemographic profiles through email and message boards. Access links to the survey routed participants to an online informed consent page before proceeding to the self-administered questionnaire. Participation was voluntary, and all participants were compensated directly through Qualtrics using standard procedures set by the panels. Incentives typically range from cash rewards to gift cards or other redeemable points. The final dataset of survey responses was prepared and deidentified (e.g., removal of IP addresses) by Qualtrics before sharing with the author. Data collection procedures occurred in 2018, after institutional review board approval, as part of a dissertation thesis.

### 2.2. Measures

#### 2.2.1. Demographics

Variables assessing age, ethno-racial identity, education, household income, and other sociodemographic information were collected.

#### 2.2.2. Perceptions of Psychological Intimate Partner Violence

Vignettes were constructed to measure perceptions of psychological intimate partner violence. They were developed out of the Lesbian, Gay, Bisexual, and Trans (LGBT) Power and Control Wheel, an adaptation from the original Power and Control Wheel that is commonly used as a psychoeducational tool in therapeutic and advocacy settings [15,41]. The use of gendered language in the initial iteration, however, defined women as victims and men as abusers. A subsequent iteration was created to challenge these assumptions, add LGBT-specific tactics of power and control, and illustrate that IPV occurs within the broader climate of heterosexism that encompasses “homophobia”, “biphobia”, and “transphobia”.

Due to the novelty of the current research, three of the eight “wheel spokes” were chosen to narratively represent psychological IPV. The process of selecting tactics occurred under expert discretion by domestic violence professionals and began by eliminating those that included a physical component. For example, the spoke on the LGBT Power and Control Wheel describing “using intimidation by instilling fear through destroying things and scary body language” was excluded. Finally, spokes focusing primarily on economic abuse were eliminated as recent research has classified this as its own form of IPV [42,43]. This approach to highlight psychological tactics was determined with the selection of the following spokes: denying, minimizing, and blaming; emotional abuse; and intimidation. Each tactic included examples that were taken verbatim from the LGBT Power and Control Wheel [41,44]. Key feedback from community professionals in the field of domestic violence was especially important and ensured that the narratives were accurately expressing the intended psychological IPV tactics and placing the characters in realistic situations to reflect details congruent with common patterns of abuse.

Three vignettes centered on separate tactics of psychological IPV occurring between women in same-gender relationships (see Table 1). Attempts were made to neutralize participant experience in reading the vignettes. Careful consideration in choosing racially and ethnically ambiguous names for the characters was intended to reduce cultural biases (e.g., views related to race, nationality, and/or ethnicity). Limiting persuasive language within the vignettes was also integral to avoid leading the participant to feel sympathy or anger towards a character.

Responses to the vignettes involved rating the behavior exhibited by each character using a 7-point slider scale with designated extremes of “not okay” and “okay”. The participants were prompted to evaluate both partners in each scenario to minimize inclinations in labelling one character as the aggressor over the other. The order in which the vignettes appeared was also randomized. Mean scores associated with the partners that perpetrated psychological IPV in each scenario were computed to assess perceptions. Higher scores indicated that the participant perceived the behavior of the character employing psychological IPV less harshly. Overall interpretations of the perceptions related to the extent to which the participants, at the observer level, identified a particular act of psychological aggression in the vignette to be problematic.

#### 2.2.3. Internalized Sexual Minority Stigma

Internalized sexual minority stigma was assessed using the Lesbian, Gay, and Bisexual Identity Scale (LGBIS). This scale was developed to evaluate eight dimensions of sexual minority identity [45]. Response options for the LGBIS are presented on a 5-point scale assessing agreement (e.g., “If it were possible, I would choose to be straight”.) and averaged across items by subscale [45]. The Internalized Homonegativity subscale, consisting of three items, demonstrated very good reliability, α = 0.83 [46].

#### 2.2.4. Perceived Sexual Minority Stigma

Perceived sexual minority stigma is a subscale from the Adapted Homophobia Scale (AHS), a measure of an external form of stigma casted upon bisexual, lesbian, and queer women [47]. Also referred to as “felt-normative stigma”, perceived sexual minority stigma describes consciousness of the cultural devaluation and negativity that exists for SMW social groups. Items of the AHS determine frequency ranging from “never” to “many times” on a 4-point scale (e.g., “How often have you felt your family was hurt and embarrassed because you are LBQ?”). Internal consistency for this subscale was acceptable, α = 0.75 [46].

#### 2.2.5. Enacted Sexual Minority Stigma

The AHS also includes a subscale measure of enacted sexual minority stigma, a different type of external stigma that captures direct experiences with rejection and exclusion occurring through acts of violence and discrimination, such as police harassment and sexual assault [47]. This subscale had very good reliability, α = 0.83 [46].

#### 2.2.6. Childhood Exposure to Domestic Violence

A subset of items taken from the Adverse Childhood Experiences (ACEs) tool has been psychometrically validated to assess abuse occurring within the household [48]. The original measure was developed by Kaiser Permanente and Centers for Disease Control and Prevention (CDC) to evaluate stressful and traumatic events during childhood [31]. Items in the ACE-abuse subscale pertain to witnessing and being victimized by physical and psychological violence before the age of 18 years. An additional ACE item on sexual victimization (i.e., “Did anyone at least five years older than you or an adult ever touch you sexually?”) was also included. For each experience, participants were asked to report on occurrence by selecting one of four answer options: (1) yes, (2) no, (3) don’t know/not sure, and (4) prefer not to say [31]. Responses were dichotomized to create groups of only participants who indicated “yes” (coded as 1) and collapsing the remaining participants into separate groups (coded as 0). On account of distinct forms of domestic violence, each childhood exposure was analyzed by item.

### 2.3. Statistical Analyses

Surveys with nonvarying responses (e.g., a participant selecting “1” for every question on the entire questionnaire) and high levels of missing data (e.g., more than half of the items in a single scale) were removed from analyses. In addition, only data from participants who provided complete responses to the vignettes assessing permissive perceptions of psychological IPV were retained for the analytic sample.

Descriptive statistics were computed for each variable, and distributions of the main variables were examined. To address the first research question, bivariate analyses were performed to assess the association between sexual minority stigma and permissive perceptions of psychological IPV. The secondary and tertiary research questions were achieved using a moderated regression approach. Centering was performed to assist with interpretations. To test the moderating role of childhood exposure to domestic violence, separate interaction terms were created for each type of sexual minority stigma with childhood exposure to domestic violence. Similarly, individual interaction terms were created for each type of sexual minority stigma paired with sexual orientation. These tests were performed with permissive perceptions of psychological IPV as the dependent variable. All analyses were conducted using IBM SPSS for Windows, Version 27.0 (IBM Corp., Armonk, NY, USA).

## 3. Results

### 3.1. Descriptive Statistics and Preliminary Analyses

Table 2 displays descriptive statistics for sociodemographic characteristics and perceptions of psychological IPV, sexual minority stigma, and childhood exposure to domestic violence variables for the total study sample (*N* = 183) as sexual orientation subgroups (bisexual = 104; lesbian = 79). Overall, most participants indicated their age to be under 35 years (*n* = 117, 67.6%) and did not identify with a Hispanic ethnicity (*n* = 154, 84.2%). Over half of the sample reported that their highest level of education was high school (*n* = 49, 26.8%) or some college without obtaining a degree (*n* = 44, 24.0%). The majority of the sample also reported an annual household income of less than $50,000 (*n* = 111, 60.7%) and assessed their economic resources as “enough to get by but no extra” (*n* = 72, 39.3%). In addition, many participants were currently in a relationship (*n* = 119), and nearly 70% (*n* = 130) of the sample did not have any children under the age of 18 years. There were significant differences between bisexual and lesbian women with regard to age and children. Specifically, bisexual women were more likely to be younger (*p* = 0.01) and have children (*p* = 0.01) compared to lesbian women.

Among the main variables of interest, bisexual and lesbian women did not significantly differ in terms of perceived sexual minority stigma, internalized sexual minority stigma, or childhood exposure to domestic violence. However, bisexual women reported significantly lower levels of enacted sexual minority stigma (*p* = 0.01) than lesbian women. Among perceptions of psychological IPV, bisexual women were more likely to negatively evaluate the perpetration of psychological IPV (*p* = 0.01). All group differences were established through comparisons of mean scores among continuous variables using *t*-tests, while categorical variables were analyzed with χ^2^ tests.

### 3.2. Association between Sexual Minority Stigma and Perceptions of Psychological IPV

Bivariate correlations were conducted to test the association of sexual minority stigma and perceptions of psychological IPV among bisexual and lesbian women. Results demonstrated that in the overall sample, there was no significant association with perceived sexual minority stigma and perceptions of psychological IPV. However, there was correlation between enacted sexual minority stigma and perceptions of psychological IPV, *r* = 0.23, *p* = 0.002, such that as levels of enacted sexual minority stigma increased, so did more permissive perceptions of psychological IPV. There was also a significant, positive correlation between internalized sexual minority stigma and perceptions of psychological IPV, *r* = 0.24, *p* > 0.001.

Additional analyses were conducted to test the same associations separately in bisexual and lesbian women (see Table 3). Correlations for perceived sexual minority stigma were significant only among lesbian women, *r* = 0.22, *p* = 0.05. Correlations for enacted sexual minority stigma were also only significant among lesbian women, *r* = 0.316, *p* = 0.01. There was, however, a significant association between internalized sexual minority stigma and perceptions of psychological IPV for bisexual women, *r* = 0.36, *p* > 0.001. This association was not significant among lesbian women.

### 3.3. Childhood Exposure to Domestic Violence as a Moderator

Multiple regression was conducted to test whether childhood exposure to domestic violence moderated the association between sexual minority stigma and perceptions of psychological IPV. No moderation effects were detected, and main effects are reported in Table 4. The following regression models included the same variables but added interaction terms using the product of the mean-centered sexual minority stigma variables with each type of childhood exposure to domestic violence, resulting in the addition of three interaction terms for each type of stigma and domestic violence variable. The centered variables were entered into separate models due to statistical power concerns of entering nine interaction terms within a single model. Analyses showed that the inclusion of such interaction terms was not significantly associated with perceptions of psychological IPV and did not significantly improve the amount of variance explained by the model. All models controlled for age, ethnicity, education, economic resources, and sexual orientation.

### 3.4. Sexual Orientation as a Moderator

To assess sexual orientation as a moderator in the relationship between sexual minority stigma and perceptions of psychological IPV, similar analytic approaches to address the second research aim were used. This included mean-centering the predictor variables before creating their respective interaction terms. Results showed that the interaction terms were not significantly associated with perceptions of psychological IPV. Moreover, the amount of variance explained by the model did not significantly improve with the addition of the interaction terms. All main effects are displayed in Table 4.

## 4. Discussion

Theoretically informed by the minority stress, social exchange, and social-ecological perspectives, this study sought to investigate the linkages between sexual minority stigma and perceptions of psychological IPV among self-identifying bisexual and lesbian women. With specific interest in the more common but less understood form of IPV that comprises psychological tactics of power and control, the results represent a contribution to the literature that has focused on physical IPV in heterosexual couples.

Results for the first research question demonstrated significant, positive associations that differed between bisexual and lesbian women. Perceived and enacted sexual minority stigmas represent external or more distal sources of stigma [5,47]. These two subtypes of external sexual minority stigma were significantly correlated with perceptions of psychological IPV. These findings somewhat align with the literature on IPV that links behavioral correlates of IPV with experiences of discrimination among bisexual and lesbian women [4]. A more recent review of sexual minority stigma measures demonstrates that the general evidence of a connection to IPV risk is mostly unclear, however [49]. It is important to distinguish that the current study did not examine IPV perpetration and victimization. The discrepancy between existing studies that show a relationship between sexual minority discrimination (or external sexual minority stigma) and IPV behavior [4,50] and the current study findings of a lack of a relationship between external sexual minority stigma and perceptions of psychological IPV may indicate differences between behaviors and perceptions that are worthy of further exploration.

A number of studies show positive relationships between internalized sexual minority stigma and physical, sexual, and psychological IPV [4,27,29]. Specific evidence of this pattern among bisexual and lesbian women demonstrates that increased levels of internalized sexual minority stigma were related to disclosing a history of IPV victimization and perpetration [4]. Indeed, the current study found that internalized sexual minority stigma was significantly and positively correlated with perceptions of psychological IPV for bisexual women. In navigating favorable and unfavorable outcomes, the significance of personal and public perceptions of psychological IPV appears to suggest a fruitful grounding of social exchange theory. While there is no evidence to speculate further, there may be nuanced effects of sexual minority stigma in assessing alternatives that may help to explain the differences between bisexual and lesbian women. Intimate relationships and access to resources, both physical and psychological, may be more limited for bisexual women who may feel doubly isolated from heterosexual and lesbian peers. Subsequent research on sexual minority stigma and potential tolerance of psychological IPV alongside personal resources is essential.

Additional research suggests that bisexual and lesbian women who may have internalized the negativity associated with their sexual minority identity may also endorse stigmatization of others [37,51]. The ensuing impact of self-hatred may shape beliefs that psychological IPV is not so problematic or intensify awareness that the characters in the scenarios may have scarcer options. This may have profound effects on bisexual and lesbian women who may tolerate their own victimization or project their internalized stigma onto their partners and other sexual minority people [51]. The psychological IPV that occurs within sexual minority couples may then keep individuals from accessing help-seeking resources especially within the heteronormative environment. The extent of exposure to domestic violence in childhood for both partners may have an additive impact and heighten tolerance for the psychological IPV portrayed in the vignettes. A more contextualized framing of IPV with concern to perceptions of tolerance and permissiveness is merited to map out the particular insidious role of internalized sexual minority stigma.

Results for the second and third research aims did not yield any significant moderation effects. This unanticipated observation suggests that, among the main variables of interest in the current study, effects of the proposed moderators were the same across groups. Pertaining to childhood exposure to domestic violence, four types of domestic violence were assessed: witnessing physical forms of domestic violence, being physically harmed, being psychologically harmed, and being sexually harmed. Existing research tends to draw upon early exposure to household violence and dysfunction as key predictors in negative health outcomes [52,53]; conceived as distal stressors, child abuse experiences, specifically sexual abuse, may have a uniquely depleting influence on permissive perceptions of psychological IPV. Unfortunately, the differentiation between bisexual and lesbian women is understudied in this literature. Current findings of bisexual women reporting significantly more psychological victimization and lesbian women reporting significantly more sexual victimization should be extended to larger study samples optimized for addressing greater nuance across groups.

Similarly, there was no indication of the moderating role of sexual orientation. This finding is rather inconsistent with the accumulating literature indicating that bisexual women experience elevated health risks and outcomes, including IPV victimization as well as IPV victimization by type [10]. The measures of sexual minority stigma in the present study did not explicitly address binegativity, however. The findings may potentially be obscured and reflect feedback that is a partial representation of their experiences. In addition, the vignettes featured women in same-gender relationships. As expected, bisexual women were less likely to report having had an adult intimate relationship with a woman. Bisexual women also tended to have lower socioeconomic statuses relative to lesbian women, where the latter reported higher income, economic resources, and education levels. Such demographic variations may be associated with differing judgements; it may be that relationship status and history rendered bisexual women to be more disapproving of the psychological IPV depicted in the scenarios.

Regarding group comparisons, lesbian women reported higher levels of perceived and enacted sexual minority stigmas, whereas bisexual women reported higher levels of internalized sexual minority stigma. There is a compelling body of literature identifying the harmful experiences with sexual minority stigma singular to bisexual women. Binary notions of sexual attraction may be affirmed by the dominant heterosexist group and sexual minority groups, vilifying bisexual women [38,39,54,55]. This independent source of stigma has been linked to perceived exclusion, hostility, and stereotyping [37,55] and may suggest that confounding variables may preclude a clearer understanding of the present findings. For example, visibility may be a relevant aspect in how stigma is perceived and experienced and, consequently, internalized. Bisexual women already contend with binegativity and monosexism [38], and there may be instances where they are more protected from external sexual minority stigma if their intimate partners are men. In this same way, lesbian women whose relationships with other women may be more visible, might face increased targeting from external sources of stigma. Factors related to sexual and romantic partners (e.g., gender) may better clarify perceptions of psychological IPV in scenarios with same- and different-gender couples.

### Study Limitations and Suggestions for Future Research

The current study had several limitations. First, the cross-sectional nature of data collection limits the examination of temporal and directional relationships. Identifying whether sexual minority stigma precedes, co-occurs, or follows permissive judgments about psychological IPV is necessary to enrich understanding of these complex variables. Second, purposive sampling procedures weaken the generalizability of the study findings; nonetheless, this strategy was advantageous for recruiting self-identified bisexual and lesbian women. Third, the measurement of permissive perceptions of psychological IPV has not been validated and requires additional testing. Prior to the study, however, the vignettes were assessed for face validity through expert review, and correlations between the three vignettes were positive and significant (*r* = 0.46, *r* = 0.38, *r* = 0.35, *p* < 0.01) in the present study. In addition, the study participants were prompted to provide qualitative information to describe why they assigned their ratings for each character. While several responses were unintelligible or left blank, the majority of the sample did attempt to explain their decisions even if they were not consistently answering for every character or scenario. Exploration of these data will strengthen the scope of interpretations of the constructed contexts of psychological IPV, conclusions drawn from the tested vignettes, and issues surrounding couple dynamics [56]. Similarly, focus groups with bisexual and lesbian women who are survivors of psychological IPV may enhance the relevancy and believability of the narratives and, subsequently, overcome barriers associated with hypothetical scenarios [56]. 

Differences in permissive perceptions of psychological IPV that may be associated with sexual minority stress is a gap that must be addressed in future research. Survey scales inquired about direct experiences of sexual minority stigma and feelings towards being a bisexual or lesbian woman; the degree to which each measure of sexual minority stigma produced stress for the participant was not evaluated. It is also important to consider that some SMW may not perceive encounters with sexual minority stigma to be stressful, but such experiences may still confer negative effects [10]. Nevertheless, potential pathways through which stress levels contribute to tolerant and permissive attitudes towards psychological IPV are key data for defining future research.

Further, the pervasive nature of binegativity as an added source of sexual minority stigma is noteworthy [5,39,55,57]; bisexual women, in comparison to lesbian women, tend to have markedly higher levels of identity uncertainty, fewer connections to the sexual minority communities, and lower levels of outness [57,58]. Given these various sources of discord, bisexual women may be less perceptive in recognizing external sexual minority stigma than lesbian women. Discrimination and harassment may still bear harm to mental and physical health, but the stigma rooted in these events may not be as apparent to bisexual women managing an array of proximal stressors. Despite theoretical and empirical support suggesting that bisexual women may be more likely to report higher levels of sexual minority stigma, lesbian women may possess greater awareness of external sexual minority stigma. Perhaps lesbian women benefit from a social connectivity that bisexual women sometimes lack, and through this, lesbian women are better equipped to label their experiences through a sexual minority stigma lens [45,59].

Future research should employ measures that consider monosexism. The external sexual minority stigma subscales in the current study contain some items that explicitly ask about discrimination, harassment, and violence that comes from “straight” people [47]. Other items do not make this exact differentiation; however, the open-endedness in the wording of such items may have primed the sample to respond similarly by considering perceived and enacted sexual minority stigmas received from heterosexual people. Therefore, the lack of sensitivity in capturing experiences of stigma from sexual minority peers is acknowledged. Special attention to stigma sources may better encapsulate the non-binary sexual orientation of bisexual women and clarify the impact of binegativity on perceptions of psychological IPV.

It will be important to consider the role of other marginalized identities that coexist with the lesbian and bisexual identity, such as racial and ethnic differences that have been highlighted in IPV research among heterosexual couples [5,60]. The ways in which a minority status outside of sexual orientation is stigmatized through discrimination, rejection, and harassment should be analyzed for a more complete understanding of perceptions of psychological IPV. For example, racism may overlap with sexual minority stigma and influence bisexual and lesbian women to rely on their intimate relationships within an environment with minimal support and alternatives. 

## 5. Conclusions

The high prevalence of psychological IPV occurs against the backdrop of a society that remains uncertain about the seriousness of nonphysical forms of IPV. As psychological IPV is complicated by the variability of personal definitions and experiences, the use of vignettes enables the evaluation of power and control tactics that may be lost in traditional measures that quantify IPV by frequency. Permissive or tolerant perspectives may be more salient in bisexual and lesbian women as they may be more adjacent sources of social support for other SMW. Such approaches may, therefore, be a valuable insight for intergroup dynamics especially when ambivalence towards psychological IPV coincides with societal norms of violence. Moreover, bisexual women may be additionally pushed to the margins by monosexism that comes from heterosexual groups and lesbian/gay groups. Health disparities work has identified bisexual women as a particular risk group among sexual minority populations. Thus, it is essential to disentangle notions of homogeneity ascribed to sexual minority status and recognize the many forms of diversity that comprise SMW. As the use of the social ecological model proliferates among IPV researchers to identify risk factors across personal, family, community, institutional, and societal levels, it is compulsory to also identify the extent of heteronormative biases and condemn victim shaming and blaming. Through this, the minority stress and social exchange lenses help to draw increased attention to population-specific variables and underlying processes that may be contributing to the disproportionately higher rates of IPV experienced by bisexual and lesbian women relative to heterosexual women. It is a blending of these approaches that will inform the next steps.

## Figures and Tables

**Table 1:** Vignette descriptions.

Tactic	Text of the Vignette
Using emotional abuse	Maria and Erica are in a committed relationship and living together. Maria makes a comment about a man’s attractiveness when they are at the mall. Erica spends the rest of the day claiming that Maria must be straight. Maria had dated a man before her relationship with Erica, and Erica brings this up. Maria feels guilty that Erica is upset.
Using isolation	Sonia and Michelle are in a committed relationship and living together. Michelle mentions her plans to grab dinner with her co-workers at a restaurant nearby after work. After the work day is over, Sonia calls Michelle and Michelle reminds her about the dinner. Sonia gets very upset, so Michelle says that she will be home in an hour. Sonia gets in her car and drives to pick up Michelle.
Denying, minimizing, blaming	Tonya and Alisha are in a committed relationship and living together. Alisha suspects that Tonya is being unfaithful. Alisha makes Tonya give her the passwords to Tonya’s social media accounts. Tonya tells Alisha that she wishes Alisha had more trust in her. Alisha tells Tonya that this should not be an issue if Tonya is loyal to their relationship and has nothing to hide.

**Table 2:** Sociodemographic and bivariate differences by sexual orientation.

Variable	Overall Sample (*N* = 183)		Bisexual Women (*n* = 104)		Lesbian Women (*n* = 79)			
	*n*	%	*n*	%	*n*	%	*p* ^a^	Cramer’s *φ*
Age ^b^	173		99		74		0.01 *	0.22
18–34 years	117	67.6	73	73.7	44	59.5		
35–54 years	42	24.3	23	23.2	19	25.7		
55+ years	14	8.1	3	3.0	11	14.9		
Education							0.27	
<High school	9	4.9	4	3.8	5	6.3		
High school diploma, GED	49	26.8	28	26.9	21	26.6		
Some college, no degree	44	24.0	31	29.8	13	16.5		
College degree	61	33.3	31	29.8	30	38.0		
Graduate degree	20	10.9	10	9.6	10	12.7		
Ethno-racial identity ^b^	182		104		78		0.55	
Hispanic	28	15.3	13	12.5	15	19.2		
White, non-Hispanic	126	68.9	74	71.2	52	66.7		
Black, non-Hispanic	20	10.9	13	12.5	7	9.0		
Other, non-Hispanic	8	4.4	4	3.8	4	5.1		
Household income							0.07	
<$50,000	111	60.7	69	66.3	42	53.2		
≥$50,000	72	39.3	35	33.7	37	46.8		
Economic resources							0.18	
Very poor, not enough to get by	10	5.5	6	5.8	4	5.1		
Barely enough to get by	22	12.0	14	13.5	8	10.1		
Enough to get by but no extra	72	39.3	46	44.2	26	32.9		
More than enough to get by	53	29.0	24	23.1	29	36.7		
Well-to-do	20	10.9	9	8.7	11	13.9		
Extremely well-to-do	6	3.3	5	4.8	1	1.3		
Relationship status ^b^	182		104		78		0.53	
In a romantic or sexual relationship	119	65.4	66	63.5	53	67.9		
Children (>18 years)							0.01 *	0.19
Yes	53	29.0	38	36.5	15	19.0		
Childhood exposure to domestic violence								
Witnessing physical victimization	51	27.9	27	26.0	24	30.1	0.51	0.05
Physical victimization	58	31.7	32	30.8	26	32.9	0.76	0.02
Psychological victimization	102	55.7	65	62.5	37	46.8	0.035 *	0.16
Sexual victimization	65	35.5	30	28.8	35	44.3	0.030 *	0.16
	*M*	*SD*	*M*	*SD*	*M*	*SD*	*p* ^a^	*d*
Perceptions of psychological IPV	2.87	1.47	2.62	1.43	3.20	1.47	0.01 *	0.40
Perceived sexual minority stigma	2.41	0.83	2.34	0.79	2.51	0.88	0.17	0.210
Enacted sexual minority stigma	1.55	0.64	1.44	0.57	1.68	0.70	0.01 *	0.39
Internalized sexual minority stigma	1.90	1.07	1.95	1.02	1.84	1.14	0.23	0.10

Note: Perceptions of psychological IPV are coded such that higher scores indicate more permissive or tolerant evaluations of perpetration behaviors. Effect sizes are reported for significant results and all main variables of interest. ^a^ Group differences between bisexual and lesbian women. ^b^ Variable with missing data where sum of respondents is displayed. * *p* < 0.05, *M* = mean, *SD* = standard deviation.

**Table 3:** Correlations between sexual minority stigma and perceptions of psychological intimate partner violence.

	(1)	(2)	(3)	(4)
1. Perceptions of psychological IPV	1	−0.17	0.08	0.15
2. Perceived sexual minority stigma	−0.06	1	0.46 **	0.22 *
3. Enacted sexual minority stigma	0.15	0.49 **	1	0.32 **
4. Internalized sexual minority stigma	0.36 **	0.03	0.19	1

Note: Lower triangle represents correlations for bisexual women and upper triangle for lesbian women. * *p* < 0.05, ** *p* < 0.01.

**Table 4:** Regression coefficients for predicting perceptions of psychological intimate partner violence.

Predictors	Attitudes towards Psychological IPV			
	***B***	***SE***	***95% CI***	***p***
Main effects				
Sexual orientation	−0.11	0.04	(−0.18, −0.04)	0.002 *
Sexual minority stigma				
Perceived sexual minority stigma	−0.06	0.02	(−0.11, −0.02)	0.01 *
Enacted sexual minority stigma	0.29	0.13	(0.04, 0.53)	0.02 *
Internalized sexual minority stigma	0.21	0.08	(0.05, 0.37)	0.01 *
Childhood exposure to domestic violence				
Witnessing physical violence	0.03	0.04	(−0.05, 0.11)	0.47
Physical victimization	−0.02	0.04	(−0.11, 0.07)	0.66
Psychological victimization	−0.01	0.04	(−0.01, 0.07)	0.76
Sexual victimization	−0.11	0.04	(−0.19, −0.003)	0.01 *
Controls				
Age	0.01	0.021	(−0.02, 0.04)	0.63
Ethnicity	−0.05	0.05	(−0.15, 0.05)	0.05 *
Education	−0.02	0.02	(−0.05, 0.02)	0.29
Economic resources	0.02	0.02	(−0.02, 0.06)	0.33

Note: *R*^2^ = 0.210; * *p* < 0.05; *B* = unstandardized coefficient; *SE* = standard error; *CI* = confidence interval.

## Data Availability

The data presented in this study are available on request from the author. The data are not publicly available due to privacy restrictions.
